# Ventrolateral prefrontal hemodynamic responses in autism spectrum disorder with and without depression

**DOI:** 10.1371/journal.pone.0256780

**Published:** 2021-08-27

**Authors:** Toshiyuki Ohtani, Akio Wakabayashi, Chihiro Sutoh, Fumiyo Oshima, Yoshiyuki Hirano, Eiji Shimizu

**Affiliations:** 1 Safety and Health Organization, Chiba University, Inage-ku, Chiba, Japan; 2 Research Center for Child Mental Development, Chiba University, Chuo-ku, Chiba, Japan; 3 United Graduate School of Child Development, Osaka University, Kanazawa University, Hamamatsu University School of Medicine, Chiba University and University of Fukui, Japan; 4 Department of Psychology, Graduate School of Humanities, Chiba University, Inage-ku, Chiba, Japan; 5 Department of Cognitive Behavioral Physiology, Graduate School of Medicine and School of Medicine, Chiba University, Cyuo-ku, Chiba, Japan; Tokai University, JAPAN

## Abstract

In clinical settings, autism spectrum disorder (ASD) with comorbid depression is often difficult to diagnose, and should be considered in treatment. However, to our knowledge, no functional imaging study has examined the difference between ASD adolescents with and without comorbid depression. We aimed to compare the characteristics and prefrontal brain function of ASD with and without depression in order to identify a biological marker that can be used to detect the difference. Twenty-eight drug-naïve adolescents with ASD (14 ASD with and 14 ASD without depression) and 14 age- and gender-matched adolescents with typical development were evaluated using several variables. These included intelligence quotient, autism quotient, depression severity using the Beck Depression Inventory 2nd edition (BDI-II), and level of social functioning using the Social Adaptation Self-evaluation Scale (SASS). In addition, frontotemporal hemodynamic responses during a verbal fluency task (VFT) were measured using functional near-infrared spectroscopy (fNIRS). The ASD group, including both of the ASD with and ASD without depression groups, showed smaller hemodynamic responses than the typical development group in portions of the left dorsolateral prefrontal cortex (DLPFC), bilateral ventrolateral prefrontal cortex (VLPFC) and anterior part of the temporal cortex (aTC) during the VFT. Moreover, the smaller hemodynamic responses in the right VLPFC during the VFT in the ASD group were associated with the worse BDI-II and SASS scores. Furthermore, the ASD with depression group showed smaller hemodynamic responses in the right VLPFC during the VFT than the ASD without depression group in a direct comparison. Adolescents with ASD showed reduced activation in broad frontotemporal regions during a cognitive task compared with those with typical development. More specifically, the right VLPFC activation reflected the level of self-estimated depression and social functioning in the ASD subjects, and could be used to discriminate between ASD adolescents with and without depression.

## Introduction

Autism spectrum disorder (ASD) is a neurodevelopmental disorder characterized by deficits in social communication and interaction, as well as restricted and repetitive patterns of behavior [1]. Depression is common among individuals with ASD, and social functioning and depression may be intimately intertwined in ASD [2]. A recent meta-analysis suggested that individuals with ASD are approximately four times more likely to experience depression than the general population, and elevated depression rates in ASD are associated with increasing age (40.2% in adult samples vs. 7.7% in samples < 18 years old) and average-to-above average IQ (52.8% vs. 12.2% when mean IQ is below average) [3]. Thus, depression is common in people with ASD, particularly for adolescents and those with average or greater intelligence; university students may be a particularly at-risk population. Hence, we focused on ASD and comorbid depression in university students. Depression can be associated with several negative outcomes, including functional impairments beyond those associated with autism. For example, the co-occurrence of depression can exacerbate impairments associated with ASD (e.g., diminished social adaptive functioning) [4, 5].

In clinical practice, accurate screening and assessment of depression in patients with ASD is often difficult because of the uncertain validity of self-report, alexithymia, poor insight common to ASD, and overlapping symptoms of ASD and depression [6]. Such overlapping features include constricted affect and social withdrawal, which can lead to diagnostic overshadowing and prevent the clinician from accurately diagnosing depression in ASD patients [6]. Moreover, social communication deficits and the inability to recognize and label emotions may prevent individuals with ASD from identifying and expressing emotional states, which can cause depressive symptoms to be overlooked by family and clinicians [7].

Previous studies that examined treatment effects for adult ASD suggested that selective serotonin reuptake inhibitors [8] and cognitive behavioral therapy [9, 10] can be effective. However, comorbid depression may affect the efficacy of these interventions. In order to accurately diagnose this commonly co-occurring disorder and thus provide more effective intervention, a biological marker that can objectively identify depression in people with ASD is required. Nevertheless, to date, there have been no comparative functional neuroimaging studies examining adolescents with ASD with and without comorbid depression.

Among neuroimaging tools, functional near-infrared spectroscopy (fNIRS) is an optical neuroimaging technique that allows the non-invasive measurement of changes in the concentrations of oxygenated and deoxygenated hemoglobin (oxy-Hb and deoxy-Hb, respectively), reflecting regional cerebral blood volume [11, 12]. In addition, fNIRS is portable, making it possible to examine subjects in a natural sitting position with high temporal resolution for brain activity during tasks. Many previous fNIRS studies used executive tasks, such as the verbal fluency task (VFT), as a cognitive task to activate regional brain function. Subjects with major depression [13, 14] and ASD [15, 16] showed reduced hemodynamic responses in the prefrontal cortex (PFC) during VFT compared with healthy controls (HCs) or subjects with typical development (TD). However, to our knowledge, there is no comparative study of ASD adolescents and ASD adolescents with comorbid depression evaluating brain activation during cognitive tasks reflecting executive function. The aim of this study was to examine the differences in brain function between adolescents with ASD and those with comorbid depression, in response to tasks reflecting executive function, using fNIRS. Based on evidence from fMRI and fNIRS studies in ASD and depression, we hypothesized that ASD adolescents both with and without depression would show reduced frontal brain activation during cognitive tasks compared to adolescents with TD, and furthermore, there would be a group difference between ASD adolescents with and without depression in the regional brain activation during the task. We also sought to reveal associations between the observed functional abnormalities in the frontal brain region and self-rated severity of depression, and level of social functioning.

## Materials and methods

### Participants

Participants included 28 adolescents with ASD (14 ASD with depression (ASD+D) and 14 ASD without depression (ASD-D)) and 14 adolescents with TD. Subjects with ASD+D and ASD-D were diagnosed according to the Diagnostic and Statistical Manual of Mental Disorders, 5th Ed. [1], Autism Diagnostic Observation Schedule Second Edition [17], and Autism Diagnostic Interview-Revised [18]. In addition, the Mini International Neuropsychiatric Interview [19] was used to confirm whether there was a current major depressive episode and screen for current and past comorbid psychiatric disorders. Subjects with ASD+D and ASD-D were recruited from students who visited the mental health counselling room in the Safety and Health Organization at Chiba University for consultation. The TD subjects were volunteer Chiba University students. ASD+D, ASD-D and TD groups were matched for gender, age, years of education, intelligence quotient (IQ), and handedness (all of the subjects were right-handed as assessed by the Edinburgh handedness inventory [20]) (Table 1). The exclusion criteria were as follows: IQ 100 or lower, history of major physical illness and/or head injury, neurological disorder, substance use, and alcohol abuse. None of the subjects were taking psychotropic medications before the study was performed.

**Table 1:** Demographic and clinical characteristics of the subjects.

	ASD+D	ASD-D	TD	p-value^a)^	Tukey’s post hoc analysis
	n = 14	n = 14	n = 14		
Age (years)	22.64 (2.41)	23.07 (2.50)	21.64 (1.15)	0.197	
Gender (male / female)	male 9 / female 5	male 9 / female 5	male 10 / female 4	0.898^b)^	
Education (years)	16.00 (1.96)	16.21 (1.76)	15.50 (1.09)	0.506	
IQ	120.86 (11.35)	118.57 (8.54)	113.21 (9.14)	0.117	
VFT performance^c)^	13.00 (4.76)	14.43 (5.69)	15.57 (5.16)	0.433	
AQ-J	36.00 (4.26)	33.57 (5.68)	13.00 (5.66)	< 0.001	ASD+D = ASD-D > TD
BDI-II	28.54 (9.48)^d)^	14.75 (5.83)^e)^	5.79 (5.07)	< 0.001	ASD+D > ASD-D >TD
SASS total score	25.79 (7.29)	29.64 (7.00)	38.64 (7.09)	< 0.001	TD > ASD-D = ASD+D
Interpersonal relation factor	10.50 (3.21)	11.64 (3.27)	16.29 (3.36)	< 0.001	TD > ASD-D = ASD+D
Motivation and interest factor	9.93 (3.89)	11.43 (3.16)	14.86 (3.66)	0.003	TD > ASD-D = ASD+D
Self-perception factor	5.36 (1.99)	6.57 (2.10)	7.50 (2.21)	0.035	TD = ASD-D > ASD+D

a) One-way analysis of variance.

b) Chi-square test. Chi square value = 0.214

c) The number of generated words during VFT.

d) Missing data for one participant.

e) Missing data for two participants.

Autism spectrum disorder: ASD, ASD+D: ASD with comorbid depression: ASD-D: ASD without depression, typically developed controls: TD, IQ: intelligence quotient, VFT: verbal fluency task, AQ-J: autism quotient Japanese version, BID-II: Beck depression inventory scale 2nd edition, SASS: social adaptation self-rating scale

Data are shown as means (SD).

Participants were provided with verbal and written information about the study, and written informed consent was obtained prior to participation. In addition, parents of minor participants were also provided with verbal and written information about the study, and written informed consent was obtained from parents when the participants were minors. This study was approved by the Research Ethics Committee of Chiba University on 27^th^ December 2017 (reference number: 29–03).

### Clinical and neuropsychological assessments

The IQ of the subjects was estimated using the Wechsler Adult Intelligence Scale 3^rd^ Edition [21]. We then used several self-rating questionnaires to evaluate the clinical characteristics of the subjects. Depression severity was assessed using the Beck Depression Inventory Scale 2^nd^ edition (BDI-II) [22], and the characteristics of ASD was evaluated using the Autistic-Spectrum Quotient Japanese version (AQ-J) [23]. The BDI-II is one of the most commonly used self-rating questionnaires for measuring the presence and severity of depression in adults and adolescents. It consists of 21 questions assessing the somatic, cognitive, and affective symptoms of depression, and the items are rated on four-point scales ranging 0–3 with a maximum total score of 63 (higher scores indicate severe depressive symptoms) [22]. The AQ-J is a self-rating questionnaire for measuring the degree to which an adult with normal intelligence has the traits associated with the autistic spectrum [23]. The AQ-J consists of 50 questions, and each of the items is scored 1 point if the respondent records the abnormal or autistic-like behavior (the total score ranged from 0 to 50) [23]. The subjects’ social functioning was evaluated using the Japanese version of the Social Adaptation Self-evaluation Scale (SASS) [24]. The SASS consists of 21 items, and the subjects were asked to answer either item 1 or item 2, in accordance with their occupational status (item 1) or other types of primary activities such as housework (item 2), and then answer the other 20 items [24, 25]. Each item was scored from 0 to 3, corresponding to the minimal and maximal social adjustment. The total score ranged from 0 to 60. Furthermore, the 20 SASS items could be classified into the following three factors using principal component analysis: (1) interpersonal relations, (2) interest and motivation, and (3) self-perception [24]. The interpersonal relations factor could be scored as the sum of the following items in SASS: “family seeking behavior,” “family relationship quality,” “gregariousness,” “relationship seeking behavior,” “external relationship quality,” “external relationship appreciation,” “social attractiveness,” and “social compliance.” The interest and motivation factors could be scored as the sum of the items: “job interest or homework interest,” “work enjoyment,” “interest in hobbies,” “quality of spare time,” “community involvement,” “social inquisitiveness,” “intellectual interest,” and “control of surroundings.” Self-perception could be scored as the sum of items other than those mentioned above.

### Verbal fluency task

VFT is a test commonly used to assess frontal lobe function and can evaluate the flexibility of cognitive function. Subjects are instructed to repeat “A / I / U / E / O” for 30 s, and then produce as many words as possible starting with the letters presented, as they are announced. Three letters are presented 20 s apart, for a total of 60 s. Finally, “A / I / U / E / O” is repeated for 70 s, and the task procedure ends. The examiner records the number of words generated every 20 s [26]. In this study, we analyzed the number of words produced and brain activity during the VFT.

### NIRS measurement

A 52-channel NIRS machine (ETG-4000, Hitachi Medical Corporation, Tokyo, Japan) was used to measure the relative changes in [oxy-Hb] and [deoxy-Hb] using two wavelengths (695 and 830 nm) of infrared light based on the modified Beer–Lambert law [27]. The distance between pairs of emitter and detector probes was set at 3.0 cm. Each measuring area between pairs of emitter and detector probes was defined as a “channel.” The region 2–3 cm beneath the scalp, which is approximately the surface of the cerebral cortex [28, 29], can be measured by NIRS. Probes were placed on the participants’ prefrontal regions and anterior parts of the temporal region. The lowest probes were placed along the T4-Fpz-T3 line in the International 10/20 system. This arrangement can measure hemoglobin levels in the bilateral prefrontal cortical areas, including the dorsolateral prefrontal cortex (DLPFC), ventrolateral prefrontal cortex (VLPFC), frontopolar cortex, and anterior part of the superior and middle temporal cortices (aTC) based on anatomical craniocerebral correction via the International 10/20 system [30]. The correspondence between NIRS channels and measurement points in the cerebral cortex is presented based on the virtual registration method [31].

The time resolution of the NIRS signal was 0.1 s. To remove any short-term motion artifacts, we used a moving average window of 5 s for the analyses. We also applied an automated method for rejecting artifacts focused on three types of noise (high frequency, low frequency, and no signal) and body movement [27].

We used the VFT as activation tasks in this study. To examine task-related activation, VFT data were analyzed using the “Integral Mode” in the ETG-4000 machine. For the integral mode, linear trend fitting was performed for the data obtained between the two baseline phases. The pre- and post-task baselines were defined as the mean values of the 10-s period just prior to the task and at the end of the 70-s post-task period in the VFT. The task period was fixed at 60 s.

### Statistical analysis

Statistical analyses were performed using PASW Statistics (version 25.0; SPSS Japan Inc., Tokyo, Japan). We used the inferior 31 channels of 52 channels for statistical analysis, similar to previous fNIRS studies [32–34]. We calculated the average changes in oxy-Hb and deoxy-Hb concentrations during the VFT (60 s) in each channel for each subject, and focused on increases in [oxy-Hb] because of its superior signal-to-noise ratio [35, 36]. First, we compared the three groups (ASD+D, ASD-D, and TD) to confirm that there were no significant differences in age, years of education, IQ and gender, using a one-way analysis of variance (ANOVA) and chi-square test, respectively. We then compared the characteristics of the three groups using one-way ANOVA to analyze differences in clinical variables. We also compared the mean [oxy-Hb] changes in the three groups using a one-way ANOVA to analyze differences in prefrontal function. Mean [Hb] data were used as the dependent variable, and diagnosis was used as the independent variable. We calculated Spearman’s rank correlation coefficients to examine the relationship between [Hb] changes during the VFT and the scores of the clinical items (BDI-II, AQ-J, three SASS factor scores, and total SASS score) when significant [oxy-Hb] differences in the channels were found between the ASD and TD groups. For [Hb] data, the NIRS signal was expressed as the product of hemoglobin concentration change and optical path length. The optical path length in an individual’s brain region is unmeasurable, and the unit of measurement is mMˑmm. We could not compare channels directly to consider the possibility that the optical path length varies at an individual level. Therefore, we performed 31 one-way ANOVAs for each channel, and the false discovery rate (FDR) approach was adopted to determine the significance level and prevent an increase in alpha error due to the use of multiple comparisons [37]. Post hoc Tukey’s tests were carried out on significant variables. Statistical significance was set at p < 0.05.

## Results

### Demographic and clinical characteristics

The demographic and clinical characteristics of the subjects are shown in Table 1. There were no significant group differences between ASD+D, ASD-D, and TD groups in age, gender, years of education, IQ, and VFT performance (Table 1). A one-way ANOVA revealed significant differences between the groups in the BDI-II score, AQ-J score, SASS factor scores of interpersonal relations, interest and motivation, and total SASS score. In a multiple comparison of these items, the scores of the ASD+D group were significantly higher than those of the TD group in the BDI-II, and lower in the AQ-J, SASS interpersonal relations and motivation and interest factor scores, and total SASS score, and those of the ASD-D group were also lower than those of the TD group in the AQ-J, SASS interpersonal relations and motivation and interest factor scores, and total SASS scores (Table 1). In addition, the scores of the ASD+D group were significantly higher than those of the ASD-D group in the BDI-II (Table 1).

### Hemodynamic response during the VFT

**Fig 1:**
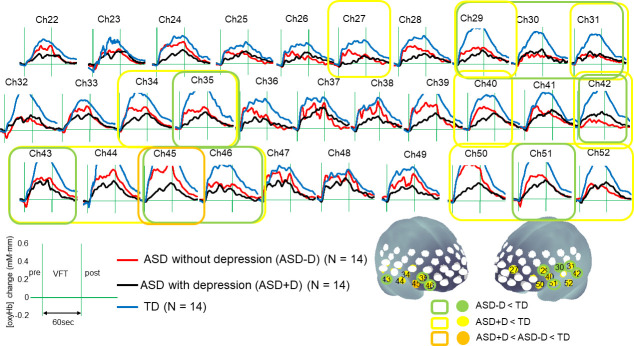
The grand-mean oxygenated hemoglobin ([oxy-Hb]) waveforms during verbal fluency task. The upper figures show the grand-averaged waveforms of [oxy-Hb] changes during the verbal fluency task in autism spectrum disorder (ASD) (red), ASD with comorbid depression (black), and typically developed (blue) adolescents. Subjects produce as many words as possible starting with the letters presented, as they are announced during the 60-sec VFT, and repeat “A / I / U / E / O pre and post the VFT. In the lower brain mapping, colored circles indicate the significant channels in one-way ANOVA (false discovery rate [FDR]-corrected p < 0.05). The results of multiple comparisons are shown by color.

The grand-mean oxy-Hb waveforms during VFT in each group are shown in Fig 1. A one-way ANOVA revealed significant differences between the groups in 15 channels (CHs 27, 29–31, 34, 35, 40, 42–46, and 50–52) located approximately in portions of the left DLPFC, bilateral VLPFC and aTC (FDR-corrected p < 0.05). In a multiple comparison of the significant 15 channels, the mean oxy-Hb changes in the ASD+D group were significantly smaller than those of the TD group in 14 channels (CHs 27, 29, 31, 34, 35, 40, 42–46, and 50–52). The mean oxy-Hb changes in the ASD-D group were significantly smaller than those in the TD group for ten channels (CHs 29‒31, 35, 42–43, 45‒46, and 51‒52). Of note, reduced activation was commonly observed in portions of the left DLPFC, bilateral VLPFC, and aTC in both groups of subjects with ASD when compared with the TD group. Furthermore, when we directly compared the difference between ASD groups, the mean [oxy-Hb] changes in the ASD+D group were significantly smaller than those in the ASD-D group in CH 45, which is located approximately in the right VLPFC. No significant differences in [deoxy-Hb] changes among the three groups were observed in any channel (FDR-corrected p > 0.05).

### Correlation between NIRS signals during VFT and demographic / clinical characteristics

**Fig 2:**
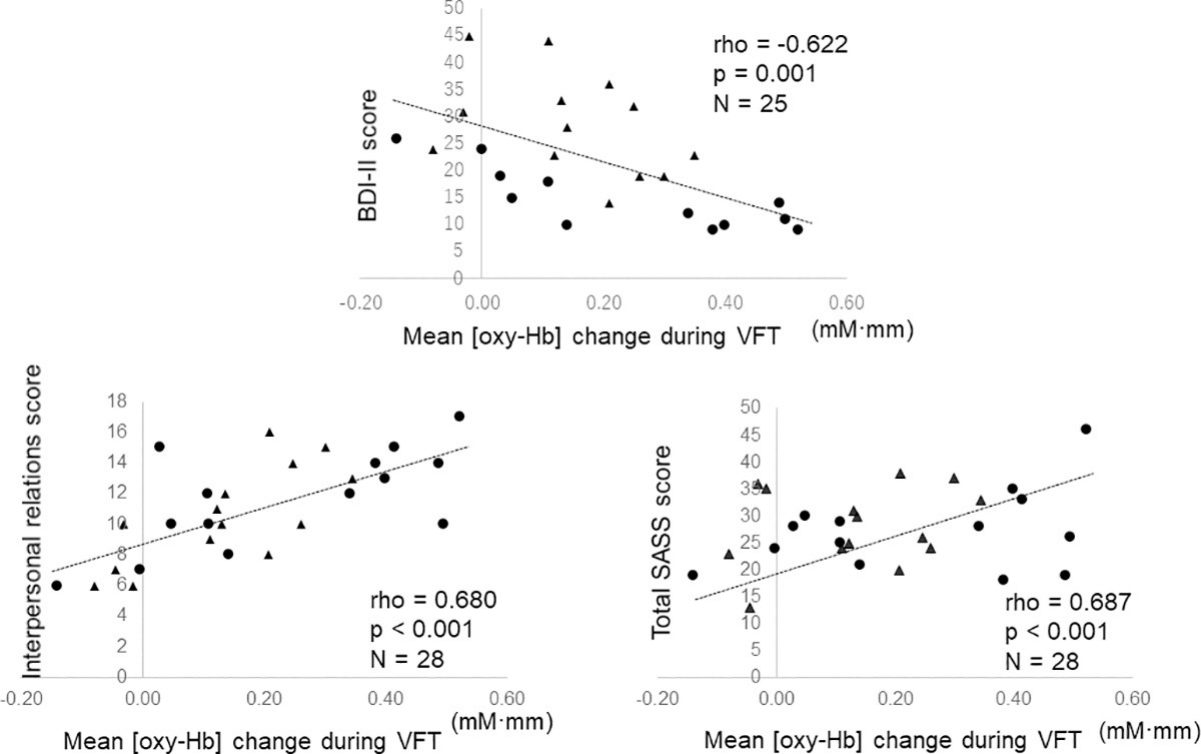
Correlation with self-rated depression severity and social adaptation including interpersonal relation and overall adaptation level in mean oxygenated hemoglobin ([oxy-Hb]) changes during the verbal fluency task. The scatter plot illustrates a typical significant channel (CH 35) in adolescents with autism spectrum disorder (ASD). The circles indicate ASD adolescents and the triangles represent ASD adolescents with comorbid depression. VFT = verbal fluency task, BDI II = Beck Depression Inventory second version, SASS = social adaptation self-rating scale, Interpersonal relation = interpersonal relation factor scores in SASS, Total SASS = total score of SASS.

We found significant negative correlations between BDI-II scores and mean [oxy-Hb] changes in all subjects with ASD in the right VLPFC (CHs 35 and 45, Spearman’s rho = -0.622 and -0.701, FDR-corrected p = 0.001, and p < 0.001, respectively) (Fig 2). We also found positive correlations between the SASS interpersonal relations factor score and mean [oxy-Hb] changes in the right VLPFC (CH 35 and CH 45, Spearman’s rho = 0.680 and 0.568, FDR-corrected p < 0.001, p = 0.002, respectively), and between the total SASS score and mean [oxy-Hb] changes in the right VLPFC (CH 35, Spearman’s rho = 0.687, FDR-corrected p < 0.001). Other variables (age, years of education, IQ, AQ-J, VFT performance) were not correlated with any CHs.

## Discussion

We measured the frontotemporal brain function in university students with ASD+D, ASD-D, and TD groups. The subjects in this study have similar backgrounds for age, years of education, and IQ. To our knowledge, this is the first study to compare the frontotemporal brain activity in ASD adolescents with and without depression using fNIRS. Comparison of frontotemporal activation between the two ASD groups (i.e. ASD+D and ASD-D) and the TD group revealed that both ASD groups exhibited significantly reduced activation in the broad frontotemporal regions including left DLPFC, bilateral VLPFC and aTC during the VFT. Of note, within the two ASD groups, activation in the right VLPFC during the VFT in the ASD+D group was reduced compared to the ASD-D group. Furthermore, worse scores on the BDI-II, SASS interpersonal relations factor, and total SASS score were associated with reduced activation during VFT in the right VLPFC in the ASD subjects. No association was observed between age, years of education, IQ, or task performance and right VLPFC activation during the tasks, suggesting that the effects of these factors on the right VLPFC activity were not significant. These findings suggest that the left DLPFC, bilateral VLPFC and aTC are involved in the pathophysiology of executive function in ASD. In addition, the right VLPFC might be the region that can distinguish adolescents with ASD+D from ASD-D, as well as reflect the severity of self-estimated depression and the social functioning in adolescents with ASD.

### Prefrontal dysfunction in ASD adolescents

Previous fNIRS studies examining prefrontal activation during VFT have reported that adult patients with ASD [15] and late-onset major depression [38] showed reduced activation in the bilateral PFC compared with HCs without task performance differences. Another fNIRS study reported region specific reduced activation in the left frontotemporal area in a comparison between ASD and TD groups [39]. The results of this study corroborate these findings, showing dysfunction during VFT in the left DLPFC, bilateral VLPFC and aTC in ASD+D and ASD-D subjects. However, another fNIRS study reported no difference of activation during VFT in a comparison between ASD and TD groups [40]. Several possible explanations were suggested for the inconsistency of results across different studies [41]. First, the ASD is made up of multiple distinct subgroups that could lead to significant population sampling differences across studies [41]. Second, the deficits in individuals diagnosed with ASD can cause different results due to relatively minor changes in administration or task formats [41]. Third, ASD causes impairments in processes that may cause unpredictable impairments in tests intended to measure higher level tasks, depending on the low-level features of specific tasks [41]. In this study, the ASD groups and TD group consisted of university or graduate school students with matched IQs and no group difference in VFT performance. The divergence between the present results and those of the previous fNIRS studies might be due to the heterogeneity in ASD or the background of the subjects including age, gender, intelligence, and comorbid psychiatric disorder. Further studies focusing on specific characteristics and backgrounds of ASD subjects may provide clues to address this divergence. In addition, as suggested in the previous study [41], specific deficits in individuals with ASD or impairments in processes in ASD can also affect the results to cause the divergence, although it would be impossible to confirm it via this fNIRS study. Further study using simple design and tasks to address the effects of the suggested factors in ASD may provide an explanation for this divergence.

### Right VLPFC hypo-activity and ASD adolescents with depression

Comparing the ASD+D and ASD-D groups, the ASD+D group showed significantly reduced activation during the VFT in the right VLPFC. In addition, the right VLPFC activation during the VFT was negatively correlated with the BDI-II score, suggesting that lower activation in the right VLPFC reflects worse self-estimated depression severity in adolescents with ASD. As stated, a previous fNIRS study reported that individuals with late onset depression showed significantly smaller activation during VFT compared with healthy controls in the bilateral PFC [38], and additionally, those with major depressive disorder showed reduced bilateral VLPFC and aTC activation during VFT [32]. The present results are partially consistent with these studies. However, when focusing on the functions that VFT requires, cognitive control processing activates rostral lateral PFC regions [42], although the present study did not show significant results in those regions. Of course, it is impossible to explain the reason for this using this study. However, it would be possible to discuss the present results focusing on the aspect of specific deficits in individuals with ASD or impairments in processes in ASD with comorbid depression. Growing evidence indicates that the right VLPFC plays an important role in downregulating emotional responses to social exclusion, and depression is accompanied by social emotional dysregulation associated with reduced lateral prefrontal engagement [43]. Thus, we speculate that ASD+D subjects, who showed reduced activation during the VFT in the right VLPFC compared with ASD-D in this study, had more severe deficits in the right VLPFC and could not regulate negative emotions (which may lead to depression). Although the mechanisms that cause a difference in right VLPFC activity between ASD+D and ASD-D could not be revealed solely by this preliminary fNIRS study, the fNIRS ability to detect depression in adolescents with ASD using biological parameters would be beneficial for clinical psychiatry.

### Right VLPFC and social functioning

Several fNIRS studies have reported an association between VLPFC activation during VFT and social functioning in depression and healthy subjects. Activation in the frontopolar and right DLPFC during the VFT was positively associated with the overall social adaptation score without an association between VFT performance and the social adaptation score in late-onset depression [38]. In addition, activation in the frontopolar and VLPFC during the VFT was also positively associated with interpersonal relationships in elderly adults; thus, the VLPFC was suggested to be relevant to interpersonal relationships in social functioning [44]. The observed positive correlation between right VLPFC activation, interpersonal relations, and overall social adaptation scores in the ASD adolescents was partially consistent with these studies, although the backgrounds of the subjects were different. The VLPFC has been suggested to be a region specialized in processing and integrating social communication information [45], and the right IFG is involved in directing social interaction [46]. Thus, VLPFC abnormalities may affect interpersonal relations and social adaptation.

## Limitations

This study had limitations. First, fNIRS measures only the brain surface activities. Thus, we could not measure activities in deep brain structures. Second, we did not measure heart and breathing rates simultaneously with fNIRS signals in this study. Thus, we could not control for changes in [oxy-Hb] and [deoxy-Hb] that were not due to functional brain activity (i.e. systemic or physiological effects on the observed results could not be excluded). Further research that examines the systemic or physiological effects on [oxy-Hb] and [deoxy-Hb] in the same study design is needed to confirm this effect. Third, the number of participants was small and consisted of university students or graduate school students only. Therefore, these findings cannot be generalized to a broader population of individuals with ASD based on this study alone. Fourth, this study did not examine TD subjects with depression. Thus, we could not directly compare ASD with depression and depression without ASD. However, we believe the observed difference between ASD+D and ASD-D in this study can contribute to better understanding for ASD with comorbid depression and provide a cue to detect it using biological markers. Fifth, we used self-rating questionnaires as clinical measures. Thus, it would be necessary to consider the validity of those subjective data in the interpretation for the observed correlations between the right VLPFC activation and clinical measures. Studies using objective assessment tools, including the caregiver report of psychopathology, are required to confirm the validity of the present results. Sixth, the lack of measures that require a simple cognitive function in this study limits the inference of neurological differences in cognitive control between the groups. It was a complex interaction between the exact sequential demands of the situation, the particular processes which are impaired, and the novelty of that situation that will determine the degree of behavioral impairment in any given individual [47]. Moreover, VFT requires various cognitive functions that may affect the observed results. In addition, the baselines we used in this study could not necessarily control for the information processing systems concerning VFT focusing on frontotemporal activities. However, the VFT and baselines we used in this study were used in the clinical settings and many previous fNIRS studies. Thus, we believe the observed results can contribute to a better understanding of ASD and ASD with comorbid depression in adolescents.

## Conclusions

Using fNIRS, we measured prefrontal hemodynamic responses in ASD adolescents with and without depression. Both of the ASD with depression and ASD without depression groups showed reduced activation in broad frontotemporal regions during a cognitive task compared with the TD group. More specifically, the right VLPFC activation reflected the level of self-estimated depression, interpersonal relations and overall social adaptation in the ASD subjects. Furthermore, reduced activation in the right VLPFC might indicate depression in adolescents with ASD. To our knowledge, this is the first study to compare adolescents with ASD with and without comorbid depression. The reason for the observed reduced activation in the right aTC during VFT in ASD with the comorbid depression group could not be explained solely by this study. Nevertheless, the findings might give a clue to understand the characteristics of ASD with depression.

## Supporting information

S1 Data (XLSX)
